# Efficiency of four solutions in removing 23 conventional antineoplastic drugs from contaminated surfaces

**DOI:** 10.1371/journal.pone.0235131

**Published:** 2020-06-22

**Authors:** Nicolas Simon, Nicolas Guichard, Pascal Odou, Bertrand Decaudin, Pascal Bonnabry, Sandrine Fleury-Souverain

**Affiliations:** 1 Pharmacy, Geneva University Hospitals and Institute of Pharmaceutical Sciences of Western Switzerland, University of Geneva, Geneva, Switzerland; 2 Univ. Lille, CHU Lille, ULR 7365 –GRITA–Groupe de Recherche sur les Formes Injectables et les Technologies Associées, Lille, France; Bhagwan Mahvir College of Pharmacy, INDIA

## Abstract

**Background:**

Residual contamination by intravenous conventional antineoplastic drugs (ICAD) is still a daily issue in hospital facilities. This study aimed to compare the efficiency (Eff_Q_) of 4 different solutions to remove 23 widely used ICADs from surfaces.

**Method and findings:**

A solution containing 23 ICADs (4 alkylating agents, 8 antimetabolites, 2 topo-I inhibitors, 6 topo-II inhibitors and 3 spindle poisons) was spread over 100 cm^2^ stainless steel. After drying, decontamination was carried out using 10×10 cm wipes moistened with 300 μL of one of the following solutions: 70% isopropanol (S1); ethanol-hydrogen peroxide 91.6–50.0 mg/g (S2); 10^−2^ M sodium dodecyl sulphate/isopropanol 80/20 (S3) or 0.5% sodium hypochlorite (S4). Six tests were performed for each decontamination solution. Two modalities were tested: a single wipe motion from top to bottom or vigorous wiping (n = 6 for each modality). Residual contamination was measured with a validated liquid chromatography with tandem mass spectrometry detection method. Solution efficiency (in %) was computed as follows: Eff_Q_ = 1–(quantity after decontamination/quantity before decontamination), as median (min–max) for the 23 ICADs. The overall decontamination efficiency (Eff_Q_) of the 4 solutions was compared by a Kruskall-Wallis test. Decontamination modalities were compared for each solution and per ICAD with a Mann-Whitney test (p<0.05).

Eff_Q_ were significantly different from one solution to the next for single wipe motion decontamination: 79.9% (69.3–100), 86.5% (13.0–100), 85.4% (56.5–100) and 100% (52.9–100) for S1, S2, S3 and S4 (p<0.0001), respectively. Differences were also significant for vigorous decontamination: Eff_Q_ of 84.3% (66.0–100), 92.3% (68.7–100), 99.6% (84.8–100) and 100% (82.9–100) for S1, S2, S3 and S4, respectively (p<0.0001). Generally, vigorous decontamination increased Eff_Q_ for all tested solutions and more significantly for the surfactant.

**Conclusion:**

Decontamination efficiency depended on the solution used but also on the application modality. An SDS admixture seems to be a good alternative to sodium hypochlorite, notably after vigorous chemical decontamination with no hazard either to materials or workers.

## Introduction

Occupational exposure to intravenous conventional antineoplastic drugs (ICADs) is a daily problem widely encountered in care settings. Traces of contamination have been found in many sectors of the drug supply chain, from industry [1] to hospitals [2,3] and to patients’ homes [4]. More recently, veterinary clinics have also been found liable to occupational exposure [5].

Since the first report [6], numerous articles have highlighted different risks, varying according to the case. For example, sudden massive contamination (e.g. vial breaking) may cause acute symptomatology [7–9], which is an uncommon event. Chronic exposure could lead to clinical or biological disorders, notably reproductive risks or cytogenetic effects [10,11].

Rapidly, professional recommendations were published [12–14], followed by institutional recommendations [15–21]. All these recommendations insist on the need to combine several protective measures. Indeed, personal protective equipments (e.g. gloves, gowns, mask…) are recommended to avoid a direct contact between the operator and the chemical hazard. Such equipments have to be combined to collective equipments (e.g. compounding isolators or laminar air-flow hoods, specific sterile medical devices for preparation or administration…) which are devoted to decrease the widepreading of the chemical contamination to the surrounding [19,21,22,23]. Nonetheless, the respect for these recommendations is not absolute and appears to differ among healthcare professionals [24]. Moreover, the use of protective tools varies according to healthcare facilities. Consequently, healthcare workers are exposed to varying levels and types of contamination.

One safety measure which must be developed is the elimination of contaminants from workplaces. This was recently mentioned in the <USP800> monograph and in the latest recommendations of the American Society of Healthcare-system Pharmacists [23,25]. Removing contamination from surfaces has been studied for more than thirty years. A recent review of the literature discussed all the decontamination strategies studied and published [26] and in what conditions: contaminants used, contamination level, surface type and operating procedure [26]. According to the <USP800>, contaminants may be removed from surfaces either by deactivation, that is to say by chemical degradation, or by decontamination, involving a physical process (i.e. desorption). Desorption strategies were studied on different antineoplastic drugs after intentional surface contamination. Some studies focused on 1 or 2 drugs [27–32], testing solutions of different natures (quaternary ammonium, biguanide, alcohol-based solutions). Others were performed on 10 drugs with a wider range of solutions (organic solvent; anionic, non-ionic or cationic surfactants; oxidant or water) [33]. Considering the different physicochemical properties of ICADs, some strategies were found to be of little efficacy (e.g. 70% isopropanol). Nonetheless, certain approaches were promising such as diluted oxidant (e.g. sodium hypochlorite) or the combination of a surfactant agent and an alcoholic solvent (e.g. sodium dodecyl sulfate/isopropanol). Even if these studies were tested on 10 compounds and have yielded interesting results, they concern only a small number of the antineoplastic drugs handled in hospital. Therefore, efficacy on numerous other drugs from different antineoplastic drug families requires evaluating.

The objective of this *in vitro* study was to assess the efficiency of four different solutions after intentional contamination on stainless steel surfaces. The originality of this study lies in the assessment of decontamination solutions and application modalities on 23 conventional antineoplastic drugs from all conventional antineoplastic drug families.

## Materials and methods

### Chemicals

The ICADs used for experiments were: 5-fluorouracil (batch# PE4SH-RM), methotrexate (batch# G07UG-01) and dacarbazine (batch# Z3J8O-SF), purchased from Tokyo Chemical Industry (Zwijndrecht, Belgium); gemcitabine (batch# A0375170), purchased from Acros Organic (Geel, Belgium); busulfan (batch# BCBN8120V), purchased from Sigma-Aldrich (Buchs, Switzerland); ganciclovir, purchased from Roche Pharma (Cymevene^®^, batch# B4091B08, Reinach, Switzerland); cytarabine (batch# 3-YFD-59-1), epirubicin (batch# 11-CGS-118-1) and topotecan (batch# 8-MSW-162-1) purchased from Toronto Research Chemicals (North York, ON, Canada); raltitrexed (batch# MG10453-29102016), pemetrexed (batch# AGN2017-685), docetaxel (batch# CS13527-18112016), paclitaxel (batch# CS22539-20112016), vincristine sulfate (batch# MC-10452-0212016), doxorubicin hydrochloride (batch# MC10454-0612016), daunorubicin (batch# MC10456-1712016), idarubicin (batch# 5-CGS-96-1), etoposide (batch# MC10457-20102016), etoposide phosphate (batch# MC305547-21082017), irinotecan hydrochloride (batch# MC20783-21082017) and fludarabine phosphate (batch# MC444607-21082017) brought from Pharmaserv (Sansstad, Switzerland). Cyclophosphamide (batch# 7F124) and ifosfamide (batch# 7H025) were obtained from Baxter EG (Endoxan^®^ and Holoxan^®^, respectively, Opfikon, Switzerland). Internal standards (IS) for the analytical assay ([^13^C^15^N_2_]-fluorouracil, batch# JA-ALS-16-130P3; [^13^C^2^H_3_]-methotrexate, batch# TM-ALS-12-134-B1; [^13^C_6_]-irinotecan, batch# TM-ALS-12-257-P1; [^2^H_8_]-cyclophosphamide, batch# LSG-ALS-12-017-P1; and [^2^H_5_]-paclitaxel, batch# TF-ALS-11-066-P1) were purchased from Alsachim (Strasbourg, France).

All solvents were mass spectrometry grade and all chemicals were obtained in the highest available analytical quality. Dimethylsulfoxide (DMSO) was purchased from Sigma-Aldrich (Buchs, Switzerland). Ammonium hydroxide, acetic acid and acetonitrile were obtained from Merck (Darmstadt, Germany). Ultrapure water (UPW) was obtained from a Milli-Q purification system from Millipore (Bedford, MA, USA).

### Preparation of working solutions

Standard stock solutions were prepared by diluting standard compounds in DMSO or UPW for pemetrexed at a concentration of 1 mg/mL and were stored at -80°C. One stock solution was prepared with:

- Group A (C_A_ = 10,000 ng/mL for each compound) comprised of 5-fluorouracil, busulfan, cyclophosphamide, cytarabine, dacarbazine, docetaxel, etoposide, etoposide phosphate, ganciclovir, gemcitabine, idarubicin, ifosfamide, methotrexate, paclitaxel, pemetrexed and raltitrexed;
- Group B (C_B_ = 50,000 ng/ml for each compound) comprised of daunorubicin, doxorubicin, epirubicin, topotecan and vincristine;
- Group C comprised of only fludarabine at a concentration of C_C_ = 100,000 ng/ml.

Internal standard stock solutions were prepared by diluting individual radiolabelled IS in DMSO at 0.1 mg/mL and also stored at -80°C.

### Contamination of surfaces

Contamination was carried out with 50 μL of a 1/50^th^ dilution of the stock solution. Thus, the intentional contaminations were 200 ng, 1,000 ng and 2,000 ng for groups A, B and C, respectively.

The 50 μL was randomly dropped on a 10×10 cm stainless steel surface and allowed to dry for 1h under a ventilated biosafety cabinet protected from light.

### Decontamination of surfaces

Decontamination followed 2 modalities: a single wipe motion from top to bottom or vigorous wiping.

Standard application consisted in a single wipe of surfaces from top to bottom.

Vigourous decontamination consisted in scrubbing surfaces energetically several times for a few seconds.

Decontamination was carried out using 10×10 cm wipes (Texwipe^TM^ 3210, ITW Texwipe, Kenersville, NC, USA) moistened with 300 μL decontamination solution. Four different solutions were tested: Solution 1: 70% isopropanol (Klercide^TM^, Ecolab, Farmham, UK); Solution 2: ethanol (91.6 mg/g) hydrogen peroxide (50.0 mg/g) (Anioxyspray^TM^, Anios, Hellemmes, France); Solution 3: 10^−2^ M sodium dodecyl sulfate:isopropanol 80:20 (home-made sterile solution) and Solution 4: 0.5% sodium hypochlorite, obtained by diluting a 3% marketed solution (Hänseler, Herisau, Switzerland) in UPW.

### Residual contamination measurement and decontamination assessment

Residual contamination was measured by applying a liquid chromatography with tandem mass spectrometry detection method developed and validated specifically to estimate surface contamination by the tested contaminants [34–36].

Briefly, sampling was performed using a polyester swab (Texwipe^TM^ 716, ITW Texwipe, Kernersville, NC, USA). Swabs were humidified with 100 μL of 75% IPA (50 μL/side) for wipe sampling [36], then introduced into 12-mL amber glass tubes containing 2-mL of desorption solution (10 mM acetic + 2% acetonitrile) and the 5 internal standards. The tube was then vortexed for a few seconds. The aqueous solution was analysed according to a previously published method [34,35].

### Statistics

All experiments were repeated 6 times and were conducted by a single operator to limit inter-individual variability of the results.

Samples were considered contaminated if at least one contaminant was quantified. For a few samples in which contaminants were unquantifiable because the signal ranged between the LOD and the LOQ, the value was expanded to the LOQ.

The contamination rate was considered to be the proportion of contaminated samples. The ability of the decontamination solution to remove chemical contamination was estimated by decontamination efficiency, defined as follows [37]:

$${\mathrm{Eff}}_{q}=1‐\frac{\mathrm{quantity}\;\mathrm{after}\;\mathrm{decontamination}\;\mathrm{procedure}}{\mathrm{quantity}\;\mathrm{before}\;\mathrm{decontamination}\;\mathrm{procedure}}\;\mathrm{for}\;\mathrm{one}\;\mathrm{contaminant}$$ Eq 1

$${\mathrm{Eff}}_{Q}=\frac{\sum {\mathrm{Eff}}_{q}}{n}\;\mathrm{for}\;\mathrm{all}\;\mathrm{contaminants}$$ Eq 2

Efficiency results are presented as medians [min–max], expressed in %. Non-parametric tests were used because of the number of samples (n < 30) and normality was unverifiable. The comparison of the overall efficiency (Eff_Q_) of the four solutions was performed with an ANOVA on ranks according to the Kruskal-Wallis method (P < 0.05). When this analysis revealed a significant p-value, contrasts were obtained with the Conover–Iman test on ranks to detect significant differences between couples of solution. The Bonferroni correction was applied on P-values to limit interpretation bias due to repeated tests. The two decontamination modalities were compared for each solution by a non parametric Mann-Whitney test (p < 0.05).

Statistics were performed in using XLSTAT^®^ for Excel^®^ (Addinsoft, Paris, France). Figures were drawn using Excel^®^ (Microsoft, Paris, France).

## Results

### Standard decontamination

The highest overall efficiency was observed with sodium hypochlorite. The medians [min–max] of overall Eff_Q_ were 79.9% [69.3–100]; 86.5% [13–100]; 85.4% [56.5–100] and 100% [52.9–100] for solutions 1, 2, 3 and 4, respectively. The Eff_q_ for each antineoplastic drug by solution are summarize in Table 1.

**Table 1:** Efficiency of the decontamination solutions on the 23 tested antineoplastic drugs on stainless steel surfaces after standard single motion. Solution 1 (S1): 70% isopropanol; Solution 2 (S2): ethanol (91.6 mg/g) hydrogen peroxide (50.0 mg/g); Solution 3 (S3): 10^−2^ M sodium dodecyl sulfate:isopropanol 80:20; Solution 4 (S4): 0.5% sodium hypochlorite aqueous solution. 5FU: 5-fluorouracil; Cyta: cytarabine; Fluda: fludarabine; Ganci: ganciclovir; Gem: gemcitabine; Mtx: methotrexate; Peme: pemetrexed; Ralti: raltitrexed; Busu: busulfan; Cyc: cyclophosphamide; Ifos: ifosphamide; Dacar: dacarbazine; Dauno: daunorubicin; Doxo: doxorubicin; Ida: idarubicin; Epi: epirubicin; EtopoP: etoposide phosphate; Eto: etoposide; Dtx: docetaxel; Pcx: paclitaxel; Irino: irinotecan; Topo: topotecan; Vin: vincristine.

	Eff_q_	5FU	Cyta	Fluda	Ganci	Gem	Mtx	Peme	Ralti
**S1**	**m**	79.9%	73.5%	86.1%	83.5%	73.6%	85.8%	100.0%	86.1%
	**sd**	36.1%	37.3%	26.8%	27.5%	37.4%	23.4%	0.0%	27.7%
**S2**	**m**	100.0%	76.1%	88.1%	94.0%	75.5%	85.7%	100.0%	100.0%
	**sd**	0.0%	25.3%	15.3%	14.6%	28.5%	13.4%	0.0%	0.0%
**S3**	**m**	100.0%	71.5%	81.0%	98.4%	71.0%	85.4%	100.0%	100.0%
	**sd**	0.0%	32.4%	31.4%	1.5%	39.4%	18.7%	0.0%	0.0%
**S4**	**m**	100.0%	98.5%	94.4%	100.0%	99.2%	100.0%	100.0%	100.0%
	**sd**	0.0%	2.6%	8.8%	0.0%	1.9%	0.0%	0.0%	0.0%
	**Eff**_**q**_	**Busu**	**Cyc**	**Ifos**	**Dacar**	**Dauno**	**Doxo**	**Ida**	**Epi**
**S1**	**m**	73.0%	77.5%	76.9%	79.7%	100.0%	75.4%	82.9%	82.5%
	**sd**	52.4%	34.5%	35.6%	34.3%	0.0%	30.1%	20.7%	16.6%
**S2**	**m**	86.5%	84.4%	83.7%	81.3%	100.0%	88.0%	66.7%	49.1%
	**sd**	28.6%	21.1%	22.8%	27.9%	0.0%	15.5%	57.7%	76.7%
**S3**	**m**	77.5%	79.6%	77.1%	74.9%	100.0%	100.0%	100.0%	82.9%
	**sd**	52.0%	31.8%	38.1%	41.6%	0.0%	0.0%	0.0%	35.8%
**S4**	**m**	100.0%	81.7%	77.5%	99.5%	100.0%	100.0%	100.0%	100.0%
	**sd**	0.0%	26.8%	35.6%	1.3%	0.0%	0.0%	0.0%	0.0%
	**Eff**_**q**_	**EtopoP**	**Eto**	**Dtx**	**Pcx**	**Irino**	**Topo**	**Vin**	
**S1**	**m**	100.0%	69.3%	81.1%	78.4%	78.8%	72.8%	100.0%	
	**sd**	0.0%	49.7%	31.4%	34.5%	35.8%	41.0%	0.0%	
**S2**	**m**	100.0%	87.1%	44.8%	64.7%	72.1%	92.6%	100.0%	
	**sd**	0.0%	19.5%	103.9%	42.3%	37.5%	16.6%	0.0%	
**S3**	**m**	100.0%	56.5%	85.8%	63.3%	78.0%	100.0%	100.0%	
	**sd**	0.0%	74.7%	21.0%	80.1%	38.7%	0.0%	0.0%	
**S4**	**m**	100.0%	100.0%	52.9%	86.7%	99.4%	100.0%	100.0%	
	**sd**	0.0%	0.0%	87.1%	18.7%	1.4%	0.0%	0.0%	

**Fig 1:**
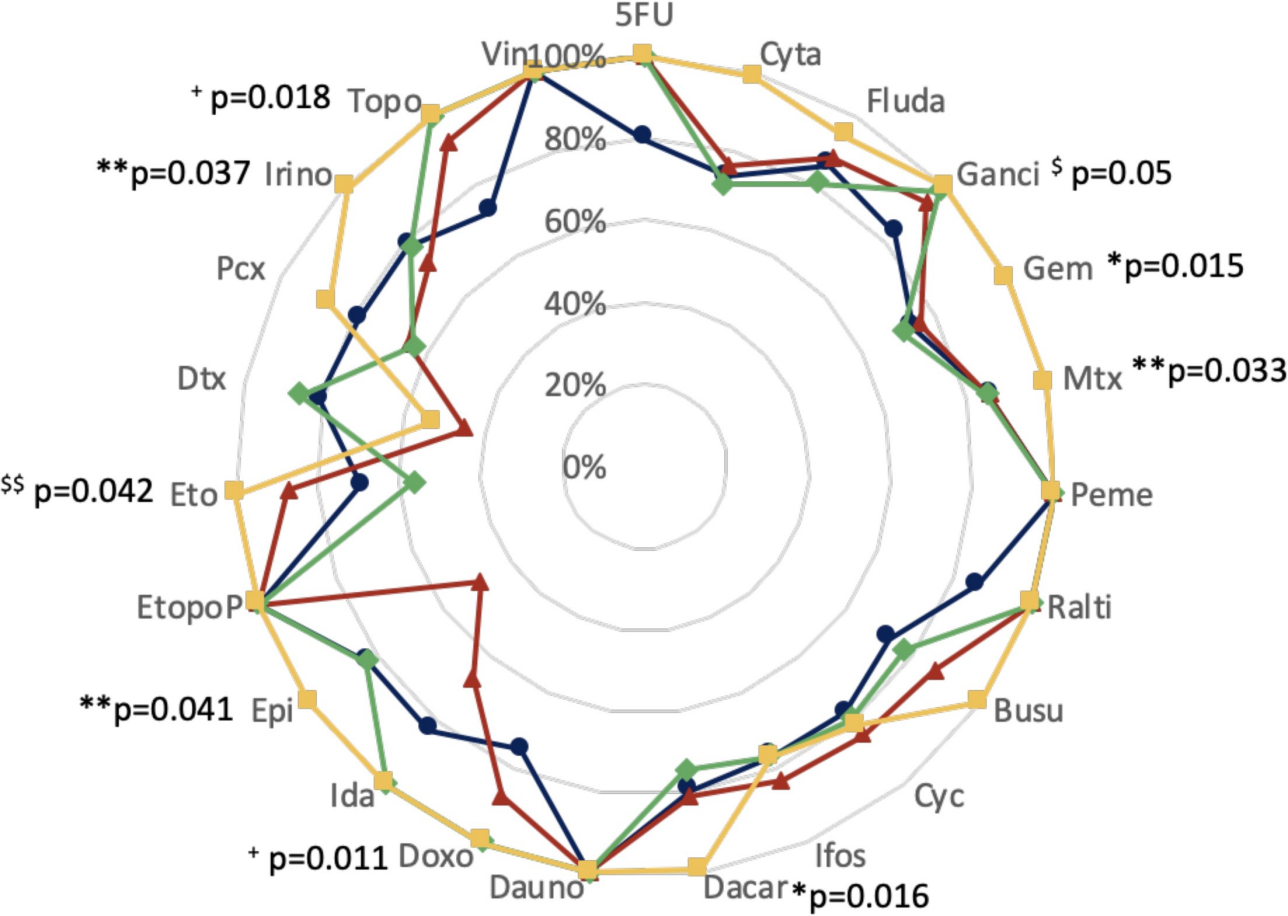
Comparison of decontamination efficiency per contaminant of four decontamination or deactivation solutions after standard single motion. Values represented are median Effq for each drug (n = 6). 5FU: 5-fluorouracil; Cyta: cytarabine; Fluda: fludarabine; Ganci: ganciclovir; Gem: gemcitabine; Mtx: methotrexate; Peme: pemetrexed; Ralti: raltitrexed; Busu: busulfan; Cyc: cyclophosphamide; Ifos: ifosphamide; Dacar: dacarbazine; Dauno: daunorubicin; Doxo: doxorubicin; Ida: idarubicin; Epi: epirubicin; EtopoP: etoposide phosphate; Eto: etoposide; Dtx: docetaxel; Pcx: paclitaxel; Irino: irinotecan; Topo: topotecan; Vin: vincristine. Blue line/circles: 70% isopropanol; red line/triangles: admixture of ethanol-hydrogen peroxide (91.6–50.0 mg/g); green line/diamonds: admixture of 10^−2^ M sodium dodecyl sulfate/isopropanol (80/20) and yellow line/squares: sodium hypochlorite. * Significant difference for solution 4 over other solutions; ** Significant difference between solutions 4 and 2; ^$^ significant difference between solutions 4 and 1 and between ^$ $^ solutions 4 and 3. ^+^ significant difference for both solutions 3 and 4 compared to others.

The lowest Eff_q_ were observed for etoposide (69.3%), docetaxel (44.8%), etoposide (56.5%) and docetaxel (52.9%) for solutions 1, 2, 3, and 4, respectively. The highest Eff_q_ was 100.0%, reached for 4, 6, 9 and 14 contaminants for solutions 1, 2, 3 and 4, respectively (Fig 1). The four solutions reached very good Eff_q_ for pemetrexed, daunorubicin, etoposide phosphate. As well as 100% efficiency, Eff_q_ reached at least 90% for 2 (ganciclovir and topotecan) and 6 contaminants (ganciclovir, raltitrexed, doxorubicin, idarubicin, topotecan and vincristine) for solutions 2 and 3, respectively. Inversely, solution 4 did not reach this threshold for 3 contaminants (cyclophosphamide, ifosfamide and docetaxel).

### Vigorous decontamination

Table 2 summarizes the Eff_q_ for each antineoplastic drug by decontamination solution. The highest overall efficiency was observed with sodium hypochlorite. The observed Eff_Q_ medians were 100%, 99.6%, 92.3% and 84.3% for S4, S3, S2 and S1, respectively. Residual contamination could be observed more frequently with solutions 1 and 2.

**Table 2:** Efficiency of the decontamination solutions on the 23 tested antineoplastic drugs on stainless steel surfaces after vigorous decontamination. Solution 1 (S1): 70% isopropanol; Solution 2 (S2): ethanol (91.6 mg/g) hydrogen peroxide (50.0 mg/g); Solution 3 (S3): 10^−2^ M sodium dodecyl sulfate:isopropanol 80:20; Solution 4 (S4): 0.5% sodium hypochlorite aqueous solution. 5FU: 5-fluorouracil; Cyta: cytarabine; Fluda: fludarabine; Ganci: ganciclovir; Gem: gemcitabine; Mtx: methotrexate; Peme: pemetrexed; Ralti: raltitrexed; Busu: busulfan; Cyc: cyclophosphamide; Ifos: ifosphamide; Dacar: dacarbazine; Dauno: daunorubicin; Doxo: doxorubicin; Ida: idarubicin; Epi: epirubicin; EtopoP: etoposide phosphate; Eto: etoposide; Dtx: docetaxel; Pcx: paclitaxel; Irino: irinotecan; Topo: topotecan; Vin: vincristine.

	Eff_q_	5FU	Cyta	Fluda	Ganci	Gem	Mtx	Peme	Ralti
**S1**	**m**	100.0%	74.9%	90.3%	84.6%	76.4%	76.4%	100.0%	94.5%
	**sd**	0.0%	13.5%	10.4%	9.6%	14.9%	29.6%	0.0%	4.2%
**S2**	**m**	95.9%	83.0%	98.2%	93.6%	85.2%	86.2%	92.3%	92.0%
	**sd**	9.1%	11.8%	4.1%	6.3%	8.8%	8.8%	4.9%	4.1%
**S3**	**m**	100.0%	84.8%	100.0%	100.0%	91.5%	87.9%	100.0%	100.0%
	**sd**	0.0%	8.1%	0.0%	0.0%	7.3%	11.4%	0.0%	0.0%
**S4**	**m**	100.0%	82.9%	91.4%	100.0%	88.2%	97.7%	100.0%	100.0%
	**sd**	0.0%	10.7%	13.7%	0.0%	7.0%	3.5%	0.0%	0.0%
	**Eff**_**q**_	**Busu**	**Cyc**	**Ifos**	**Dacar**	**Dauno**	**Doxo**	**Ida**	**Epi**
**S1**	**m**	87.2%	80.6%	81.9%	84.3%	100.0%	66.0%	72.8%	74.4%
	**sd**	10.8%	13.3%	12.9%	13.6%	0.0%	26.0%	26.1%	14.3%
**S2**	**m**	91.5%	94.9%	93.5%	93.8%	97.8%	95.3%	75.8%	83.2%
	**sd**	8.6%	3.7%	4.5%	5.7%	5.4%	7.8%	12.6%	15.1%
**S3**	**m**	94.6%	93.7%	93.7%	92.5%	100.0%	100.0%	86.1%	100.0%
	**sd**	5.9%	3.5%	3.6%	4.2%	0.0%	0.0%	31.0%	0.0%
**S4**	**m**	94.8%	89.8%	89.9%	100.0%	100.0%	100.0%	91.0%	100.0%
	**sd**	5.9%	7.0%	7.7%	0.0%	0.0%	0.0%	20.1%	0.0%
	**Eff**_**q**_	**EtopoP**	**Eto**	**Dtx**	**Pcx**	**Irino**	**Topo**	**Vin**	
**S1**	**m**	100.0%	86.3%	82.6%	69.9%	82.5%	92.9%	100.0%	
	**sd**	0.0%	9.4%	17.9%	31.8%	9.1%	9.4%	0.0%	
**S2**	**m**	100.0%	96.6%	74.0%	68.7%	89.3%	92.3%	95.9%	
	**sd**	0.0%	2.7%	20.3%	62.8%	8.9%	6.1%	10.1%	
**S3**	**m**	100.0%	91.2%	97.9%	99.0%	100.0%	99.6%	100.0%	
	**sd**	0.0%	6.4%	3.0%	2.5%	0.0%	0.5%	0.0%	
**S4**	**m**	100.0%	100.0%	85.9%	84.8%	100.0%	100.0%	100.0%	
	**sd**	0.0%	0.0%	16.1%	19.1%	0.0%	0.0%	0.0%	

**Fig 2:**
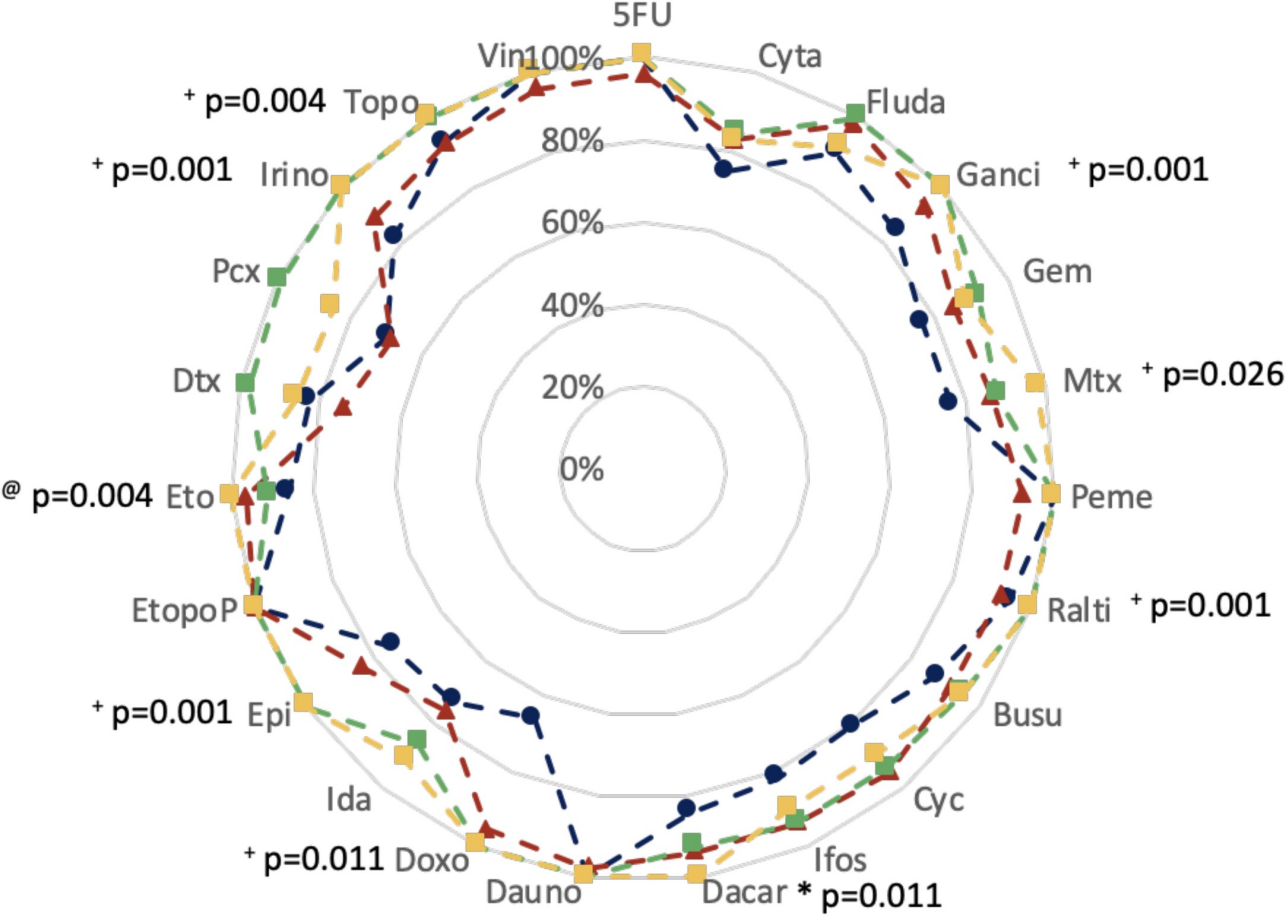
Comparison of the decontamination efficiency per contaminant of four decontamination or deactivation solutions after vigorous decontamination. Values represented are median Effq for each drug (n = 6). 5FU: 5-fluorouracil; Cyta: cytarabine; Fluda: fludarabine; Ganci: ganciclovir; Gem: gemcitabine; Mtx: methotrexate; Peme: pemetrexed; Ralti: raltitrexed; Busu: busulfan; Cyc: cyclophosphamide; Ifos: ifosphamide; Dacar: dacarbazine; Dauno: daunorubicin; Doxo: doxorubicin; Ida: idarubicin; Epi: epirubicin; EtopoP: etoposide phosphate; Eto: etoposide; Dtx: docetaxel; Pcx: paclitaxel; Irino: irinotecan; Topo: topotecan; Vin: vincristine. Blue line/circles: 70% isopropanol; red line/triangles: admixture of ethanol-hydrogen peroxide (91.6–50.0 mg/g); green line/diamonds: admixture of 10^−2^ M sodium dodecyl sulfate/isopropanol (80/20) and yellow line/squares: sodium hypochlorite. ^+^ significant difference for both solutions 3 and 4 compared to others.; ^@^ significant differences between the 4 solutions; * significant difference of solution 4 over other solutions.

The four solutions yielded the same decontamination efficiency for cytarabine, daunorubicine, vincristine and 5-fluorouracil. Oxazophosphorines were removed more efficiently, notably with solutions 2, 3 and 4. Solution 3 had better Eff_q_ on both docetaxel and paclitaxel (Fig 2).

Eff_q_ comparison of the four solutions using the Kruskall-Wallis test revealed some significant differences for doxorubicin (S1 = 66.0%; S2 = 95.3% vs. S3 = 100%; S4 = 100%; p = 0.011), epirubicin (S1 = 74.4%; S2 = 83.2% vs. S3 = 100%; S4 = 100%; p = 0.001), ganciclovir (S1 = 84.6%; S2 = 93.6% vs. S3 = 100%; S4 = 100%; p = 0.001), irinotecan (S1 = 82.5%; S2 = 89.3% vs. S3 = 100%; S4 = 100%; p = 0.001), methotrexate (S1 = 76.4%; S2 = 86.2% vs. S4 = 97.7%; p = 0.026), raltitrexed (S1 = 94.5%; S2 = 92.0% vs. S3 = 100%; S4 = 100%; p = 0.001) and topotecan (S1 = 92.9%; S2 = 92.3% vs. S3 = 99.6%; S4 = 100%; p = 0.004). For these contaminants, the Conovar-Iman analysis revealed contrasts between couples of solutions: solutions 3 and 4 versus solutions 1 and 2. A significant difference was observed on etoposide between the four solutions (S1 = 86.3% vs. S2 = 96.6% vs. S3 = 91.2%; S4 = 100%p = 0.001). Finally, solution 4 had a greater effect than other solutions on dacarbazine (S1 = 84.3%; S2 = 93.8%; S3 = 92.5% vs. S4 = 100%; p = 0.002).

In this context, decontamination efficiency also depended on the tested contaminant (Fig 2). The lowest Eff_q_ were observed for doxorubicin (66.0%), paclitaxel (68.7%), cytarabine (84.8% and 82.9%) for solutions 1, 2, 3, and 4, respectively. The highest Eff_q_ (i.e. 100%) was observed on 5, 1, 11 and 13 contaminants for solutions 1, 2, 3, and 4, respectively. An Eff_q_ of 100% was observed on etoposide for the four tested solutions. Solution 1 reached an Eff_q_ ≥ 90% for fludarabine, raltitrexed and topotecan. For solution 2, this threshold was attained except for cytarabine, gemcitabine, methotrexate, epirubicin, docetaxel and paclitaxel. The Eff_q_ for solution 3 was lower than 90% for cytarabine, methotrexate and idarubicin. In the case of solution 4, this threshold was not reached for cytarabine and gemcitabine, nor for docetaxel and paclitaxel. Both cyclophosphamide and ifosfamide were decontaminated with an Effq of 89.8 and 89.9%, respectively.

### Comparison of decontamination modalities

**Fig 3:**
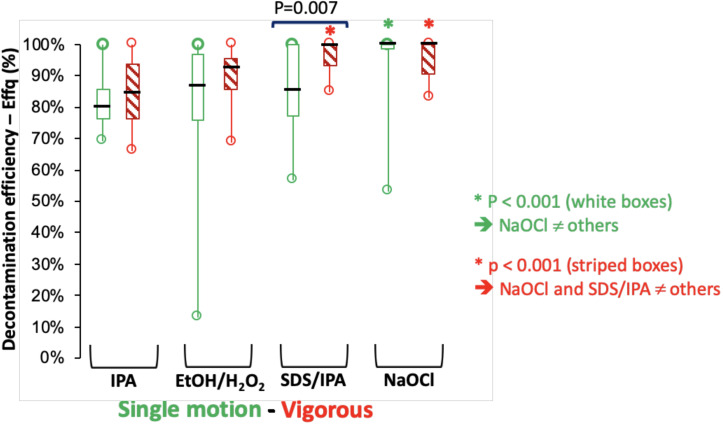
Comparison of the overall efficiency of four decontamination or deactivation solutions according to their application modalities. White boxes represent standard decontamination and striped boxes represent vigorous decontamination. The solutions compared are 70% isopropanol (IPA), an admixture of ethanol-hydrogen peroxide (91.6–50.0 mg/g), an admixture of 10^−2^ M sodium dodecyl sulfate/isopropanol (80/20) and sodium hypochlorite (NaOCl). Significant differences are observed for single standard motion between NaOCl and the other solutions and for vigorous application for both NaOCl and SDS/IPA compared to the two other solutions.

After standard application of the solutions (Fig 3), a significant difference was observed for solution 4 compared to the others (p < 0.001) according to the Mann-Whitney test. After vigorous application, Eff_Q_ were 84.3% [66.0–100]; 92.3% [68.7–100]; 99.6% [84.8–100] and 100% [82.9–100] for solutions 1, 2, 3 and 4, respectively (Fig 3) with a significant difference for both solutions 3 and 4 compared to solutions 1 and 2 (p < 0.001). Vigorous application significantly increased the Eff_Q_ of solution 3 compared to the standard application (p = 0.007). This result was not noted for the other solutions.

## Discussion

Many products have been marketed for use in compounding units to remove chemical contamination by ICADs. Despite these, contamination is still present. [2,38]. In compounding units, both containment primary and secondary engineering controls (respectively C-PEC and C-SEC) are contaminated, which means that safety measures have to be improved. As mentioned in the <USP800> monograph, the removal of chemical contaminants may be obtained by two means: deactivation or decontamination [25]. Among the solutions tested in this study, sodium hypochlorite and hydrogen peroxide can deactivate conventional antineoplastic drugs as they are good oxidisers. As the oxidation of contaminants may generate degradation compounds, these were not detected by our assay and probably persist on the surface. Moreover, such oxidants may also act on the surfaces of isolators or BSC and may degrade them over time.

The originality of this study lies in the range of drugs studied whereas in literature the majority have been limited to few markers, sometimes up to 10 contaminants in the same contamination [26]. New data is therefore accessible even as regards oxidisers that have been studied thoroughly in the past.

Our study confirms that sodium hypochlorite has good overall efficiency compared to other solutions after standard application, especially on antimetabolites, anthracyclines, topo-I and topo-II inhibitors. These results are consistent with previously published results [27,29,33]. Indeed, 0.5% sodium hypochlorite was successfully tested on stainless steel surfaces on a miscelleanous of 10 antineoplastic drugs with an Eff_Q_ of 97.5% [33]. In the the other studies, Adé et al., and Hon et al. have tested only cyclophosphamide, with an Eff_q_ of 99.74% and 96.62%, respectively. Our result observed on paclitaxel shows that 0.5% sodium hypochlorite may be efficient, as it was previously demonstrated by Lee et al [31]. In this study, the lowest effect is observed on taxanes, with a marked effect on docetaxel, which explains the difference in the Eff_q_ range comparatively to the study of Queruau-Lamerie et al.

This study also confirms that 70% isopropanol is a poor decontaminating agent, as has already been observed [33]. In a previous literature review, the Eff_Q_ of 70% isopropanol on stainless steel surfaces reached a mean of 80.6%, [26] compared with 79.9% in our study. Its decontamination effect on taxanes is higher than NaOCl, probably because it is an organic solvent capable of removing these contaminants by physicochemical affinity.

Two other decontamination solutions were tested. Marketed biocide combining an oxidiser and ethanol proved to be a poor chemical decontaminant. Indeed, if a threshold of 90% is supposed to define a decontaminant enough efficient [39], the solution 2 (admixture of EtOH/H_2_O_2_) reached such a value for only 8 contaminants. This solution was chosen because it is used in practice as a disinfectant in many compounding units. Solution 2 was effectively previously tested in a real-life study and compared to solution 3, showing a lower efficiency [40]. As the formulation of disinfectants can vary, our results should not be generalised. It is however advisable to test whether a disinfectant is a good chemical decontaminant before using it [39].

The admixture of SDS/IPA tested here was previously assessed [28,33] and tested on different drugs, demonstrating a difference in Eff_q_ depending on contaminant as was also observed in our study. In the study of Queruau-Lamerie et al., such solution reached an Eff_Q_ of 87.5% on stainless steel surfaces [33], closed from our results (i.e. 85.4%) and also shown variable Eff_q_ depending on the contaminant.

Vigorous application tended to increase the Eff_Q_ for all solutions, but the effect was statistically significant for only solution 3. Therefore, both disinfectant solutions proved to be the worst chemical decontaminants when compared to SDS/IPA and NaOCl in this particular case. These two solutions had comparable Eff_Q_ after vigorous application. The biggest difference between the two solutions was certainly due to the greater effect of SDS/IPA on the two taxanes, indicating the advantage of surfactant solutions in removing lipophilic and hydrophilic drugs in the same action. A significant effect of vigorous application was found only for SDS/IPA in this study, implying the importance of application modalities (i.e. the human factor) in the effectiveness of the decontamination process. This seems logical as the contaminants to be removed have to be incorporated into the formed micelles and this requires a mechanical action which may indicate variability in decontamination efficacy between operators: variability in the strength and energy applied during the decontamination process. SDS/IPA solution was previously tested during two studies in real conditions [37,40]. Among the differences between these two studies, a human factor was suspected to explain the difference in the results, notably by the application modalities of the decontamination solution [26]. It is therefore important to consider the training of the operators involved in decontamination to limit this potential interindividual factor as much as possible.

The handling of ICADs necessitates both sterility [41] and safety in care facilities [21]. The <USP800> monograph distinguished two situations relative to duality in the context of sterile compounding: removing microorganisms with a cleaning or a disinfectant solution and removing chemical contaminants using a deactivation or a decontamination solution [25]. In medical wards, this problem has to be analysed in just the same way, as antimicrobial solutions are used there but their decontamination potential is not known. In a previous work, the performance of decontamination solutions was assessed according to three characteristics: overall efficiency (Eff_Q_), the number of tested contaminants and the risk of using a decontamination solution [39]. For routine decontamination, two solutions among those tested here have been shown to be the best decontaminants against the whole range of contaminants tested. Therefore some critical points have to be taken into account like the oxidising character of NaOCl and the risk of deposing organic residue at the end of the decontamination process. All these points have previously been discussed [39]. The last important point with regard to our results is the human factor implicated in the whole decontamination process. Indeed, this study has highlighted that decontamination modalities can condition decontamination efficacy just as in other studies that have already explored factors liable to modify it [26]. This study also underlines the importance of operator training so as to standardise decontamination and establish the most efficient procedure which takes into account the duality of handling hazardous and sterile drugs. A new pedagogical tool could be formulated to this end [42].

## Conclusion

This study, assessing four solutions, concludes that sodium hypochlorite is the best decontaminant after standard application although an admixture of surfactant agent and isopropanol gave the same results after vigorous application on 23 contaminants. Decontamination efficiency depends on the solution used but also on application modality. An SDS admixture seems to be a good option, notably for vigorous chemical decontamination without any hazard to either materials or workers.
